# Association between transplant glomerulopathy and graft outcomes following kidney transplantation: A meta-analysis

**DOI:** 10.1371/journal.pone.0231646

**Published:** 2020-04-28

**Authors:** Gábor Kovács, Giovanna Devercelli, Tamás Zelei, Ishan Hirji, Zoltán Vokó, Paul A. Keown

**Affiliations:** 1 Syreon Research Institute, Budapest, Hungary; 2 Shire, a Takeda company, Lexington, Massachusetts, United States of America; 3 Center for Health Technology Assessment, Semmelweis University, Budapest, Hungary; 4 Syreon Corporation, Vancouver, British Columbia, Canada; 5 University of British Columbia, Vancouver, British Columbia, Canada; Imperial College Healthcare NHS Trust, UNITED KINGDOM

## Abstract

Transplant glomerulopathy (TG), a morphological lesion associated with confluent mechanisms of endothelial injury of renal allografts, may provide a viable predictor of graft failure. This systematic literature review and meta-analysis were performed according to the PRISMA statement to examine evidence describing the association between TG and graft loss or failure and time to these events. The literature review was conducted using the Scopus, EBSCO, and Cochrane Library search engines. Hazard ratios, median survival times, and 95% confidence intervals (CIs) were estimated to evaluate graft survival in the total population and prespecified subgroups. Meta-regression analysis assessed heterogeneity. Twenty-one publications comprising 6,783 patients were eligible for data extraction and inclusion in the meta-analysis. Studies were highly heterogeneous (I^2^ = 67.3%). The combined hazard ratio of graft loss or failure from random-effects meta-analysis was 3.11 (95% CI 2.44–3.96) in patients with TG compared with those without. Median graft survival in patients with TG was 3.25 (95% CI 0.94–11.21) years—15 years shorter than in those without TG (18.82 [95% CI 10.03–35.32] years). The effect of time from transplantation to biopsy on graft outcomes did not reach statistical significance (p = 0.116). TG was associated with a threefold increase in the risk of graft loss or failure and a 15-year loss in graft survival, indicating viability as a surrogate measure for both clinical practice and studies designed to prevent or reverse antibody-mediated rejection.

## Introduction

Kidney transplantation offers an important opportunity to improve patient survival, quality of life, and societal functioning for patients with end-stage renal disease [1–4]. Sequential advances in transplantation biology, medicine, surgery, and pharmacology have enhanced the safety and early success of transplantation [5–8], with functional graft survival now exceeding 90% at 1 year post-transplant in Australasia, Europe, the United Kingdom, and the United States; but deeper analysis of these data shows that only 50% of all grafts survive for >10–15 years [9]. Because of the complexity of long-term trials, computational modeling has been used to identify principal risks for chronic graft failure [10]. Precision medicine strategies have been proposed to minimize these factors, and personalized care models proposed to predict and prepare for safe transition to dialysis [11, 12]. Despite these advances, premature graft failure remains a major risk to patient health and a barrier to maximizing the utility of transplanted kidneys [12].

Endothelial injury (EI) is a principal pathogenic mechanism of premature graft failure, and may reflect the confluence of both immune and nonimmune factors, which include alloantibodies, various autoantibodies, cell-mediated immunity, thrombotic microangiopathy, or chronic hepatitis C [13]. Antibody-mediated rejection (AMR), currently the leading individual cause of graft loss [14–16], is characterized by donor-specific antibodies (DSAs) that bind to human leukocyte antigens (HLAs) or other allogeneic targets on the graft. Antibodies to overt or cryptogenic autoantigens, including MHC class I chain-related genes A and B, vimentin, LG3, and other targets, may cause or amplify this response [17–19], causing a complex cascade of complement activation, microvascular injury, inflammation, and tissue remodeling and resulting in reduced graft function and proteinuria [13, 20, 21]. While less common, cell-mediated rejection and thrombotic microangiopathy (often related to calcineurin inhibitor use) are well-described antecedents of EI, and the glomerular lesions of hepatitis C may mimic or amplify the injuries triggered by these or other causes [22].

EI resulting from these factors is phenotypically heterogeneous—it may occur throughout the transplant course; and presentation may range from primary graft dysfunction to acute and fulminant graft injury to the more common and often initially asymptomatic chronic form, with the characteristic histological picture of chronic active AMR [21]. The *de novo* development of antibodies to donor HLA or other targets may inform this progression [23], but the level of evidence in predicting chronic graft loss is low [24].

Studies of novel therapeutic interventions designed to arrest or reverse this graft injury require robust predictive markers of graft failure [25]. Transplant glomerulopathy (TG) is one of the most important histological markers associated with EI [26]; it is a common and discrete morphological lesion resulting from chronic active and repeated endothelial damage. TG is characterized by the duplication of glomerular basement membranes, mesangial matrix expansion, and mesangial cell interposition that classically result from chronic recurring EI mediated by DSAs or the other immunological mechanisms outlined [13]. TG may be detected on biopsy in patients with unresolved EI or AMR months or years before graft dysfunction, and is an important factor in predicting graft loss that would necessitate return to dialysis or re-transplantation [13, 14]. This analysis was conducted to examine all relevant evidence to more precisely quantitate the risk of, and time to, graft loss following the diagnosis of TG on biopsy, to consider this as a robust end point for interventional studies, and to guide care plans for safe and efficient return to dialysis where treatment is ineffective.

## Materials and methods

A systematic literature review was conducted according to standardized Cochrane methods [27] to identify published studies of kidney transplantation that evaluated the association between TG and graft loss or failure. Searches were performed via the Scopus, EBSCO, and Cochrane library search engines and included all studies published until July 4, 2019 (search terms, individual bibliographic databases, and the search engine approach are included in S1 and S2 Tables). Two independent expert reviewers (GK and TZ) evaluated all abstracts and articles identified for full-text review that met the prespecified eligibility criteria as outlined per the relevant PICOTS (patient, intervention, comparator, outcome, timing, and setting) elements as defined in the CHARMS (checklist for critical appraisal and data extraction for systematic reviews of prediction modeling studies) methodology [28]. Publications were included for full-text review if they reported studies of patients with a kidney transplant who had a diagnosis of AMR or glomerulopathy not caused by ischemia or other defined immune glomerular disease, and included estimates of the association between TG and graft failure. Studies that were not published in English, included <10 patients, had no publication abstract available for evaluation, or duplicated prior published data were excluded. Although a potential source of bias, studies with <10 patients were excluded because they were typically case series without the depth or balance of information required for formal meta-analyses. Data extraction was undertaken by a single reviewer and was independently verified by a second reviewer. Discrepancies were resolved together by both reviewers and a third project member (ZV). Extracted data included the study population under investigation; time period of data collection and reporting; special subgroups or populations studied; sample size; biopsy type (for cause or per protocol); time from transplant to biopsy; whether or not time of biopsy was reported as the start of follow-up; median survival times; and rates of graft loss or failure, reported hazard ratios (HRs), and 95% confidence intervals (CIs).

Meta-analysis was performed using HRs to measure the association between TG and graft survival to summarize the data extracted from the included studies. Studies were excluded from the meta-analysis if they did not (1) include a quantitative comparison of graft loss or failure between patients with and without TG; (2) report on graft follow-up initiated at the time of biopsy; or (3) include graft outcome data from which an HR could be calculated. If HRs were not presented in the article, they were derived from Kaplan-Meier curves using plot-digitizing applications (WebPlotDigitizer, https://automeris.io/WebPlotDigitizer/, Ankit Rohatgi, Austin, TX; and DataThief, https://datathief.org/, B. Tummers) or from event-free probabilities at fixed time points if Kaplan-Meier curves were not presented. In all cases, constant hazards were estimated in both groups (i.e. patients with and without TG). The lower and the upper borders of the 95% CI were calculated based on the standard error of the HR. This standard error was derived from the number of patients suffering an event during the follow-up period [29].

Additionally, median overall graft survival times were derived in three ways in the following order: (1) extracted from studies if they were published; (2) calculated based on digitized Kaplan-Meier curves if patient group follow-up was beyond the median survival time; or (3) estimated by fitting a Weibull model on the digitized Kaplan-Meier curve reported [30]. Standard errors of the logarithm of the median survival times were estimated by the method described by Zang *et al*. [31]. Reasons for excluding articles are shown in S3 Table.

Two major outcomes of interest were included in the meta-analysis: Graft loss was defined as the cessation of graft function or death, and graft failure was defined as either graft loss or some laboratory change related to graft dysfunction (such as doubling of serum creatinine levels or reaching ≥150% of baseline value at time of biopsy, glomerular filtration rate (GFR) <15 mL/min/1.73 m^2^ or >50% reduction beyond 1 year). Death-censored graft loss and death-censored graft failure data were extracted where available.

Random-effects meta-analysis was used if heterogeneity was identified between the individual study estimates, as determined by the value of the heterogeneity χ^2^ test and I^2^ statistics. Because of the skewed distribution of median survival time, the analysis was performed after logarithmic transformation.

Meta-regression was used to evaluate whether key covariates available from the data, including time from transplantation to biopsy, patient age, or sex distribution, could explain any observed heterogeneity of the effect of TG. Additional sensitivity analyses evaluated the association between TG and graft outcomes within prespecified subgroups of the studies. STATA SE 15.0 (StataCorp. 2017. Stata Statistical Software: Release 15. College Station, TX: StataCorp LLC) was used to perform the analyses. Publication bias was assessed using Egger’s test and funnel plots. A risk of bias assessment was performed through the evaluation of study quality using the Selection and Outcomes domains of the Newcastle-Ottawa Scale [32]. Reporting of results from the systematic literature review and meta-analyses follow the PRISMA Statement (S1 File) [33].

## Results

### Literature search

**Fig 1:**
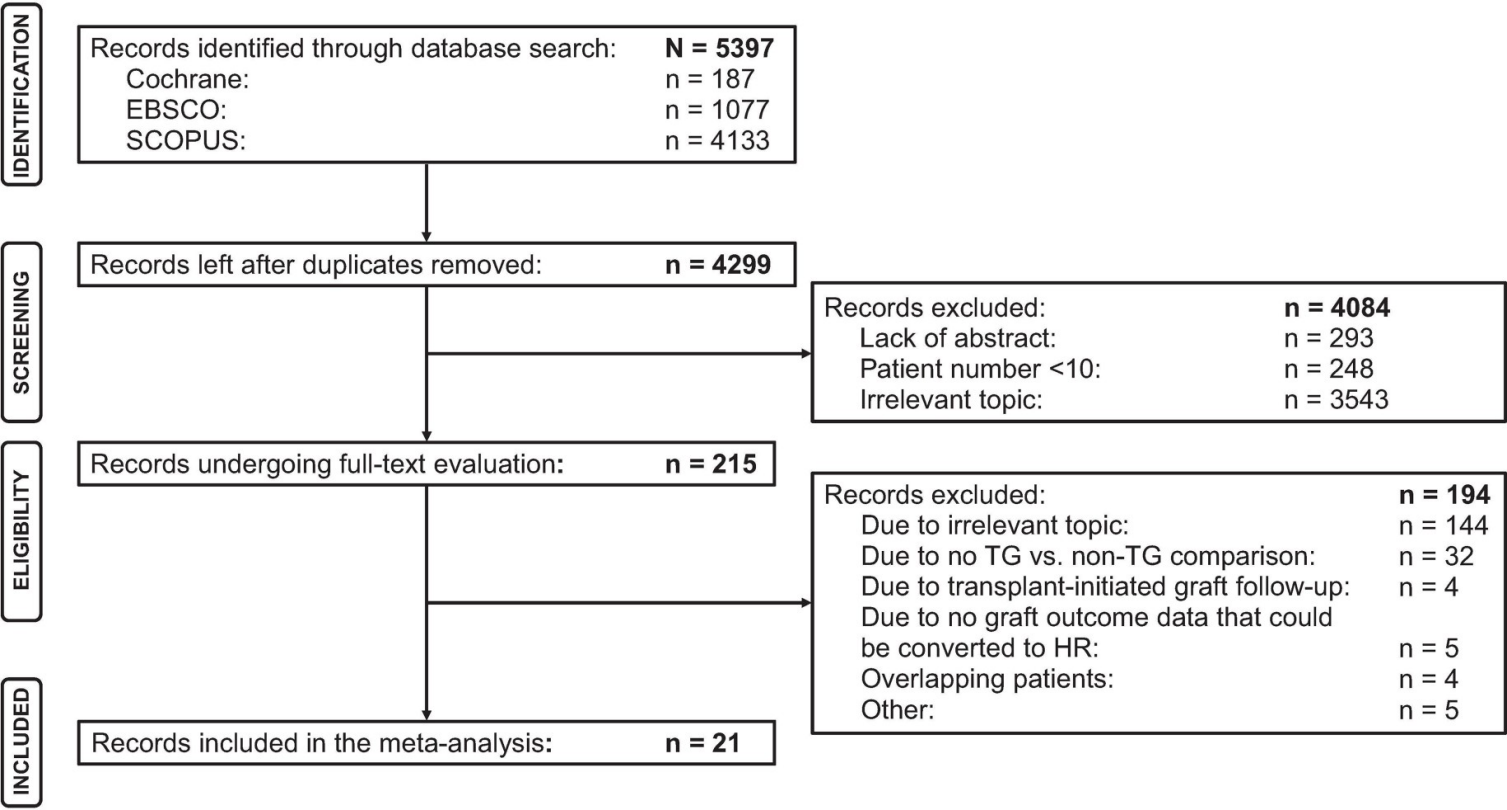
The inclusion and exclusion process of publications in the systematic review and meta-analysis. HR, hazard ratio; TG, transplant glomerulopathy.

The search strategy yielded 5,397 publications, of which 4,299 abstracts were screened and 215 published articles were reviewed. After full-text review, 194 publications were eliminated based on the eligibility criteria, leaving a total of 21 studies comprising 6,783 patients for potential inclusion in the meta-analysis [26, 34–50]. Because not all subgroups of patients reported were relevant for the analysis, data for 5,833 patients were ultimately included. The PRISMA (Preferred Reporting Items for Systematic Reviews and Meta-Analyses) flow diagram of publication screening and eligibility assessment with all exclusion categories summarized is presented in Fig 1.

### Characteristics of studies included in the analysis

The 21 studies included in the meta-analysis, shown in Table 1, report data published between April 2000 and April 2019 by transplant groups in 12 countries, including Australia, Austria, Belgium, Brazil, Canada, China, France, Germany, the Netherlands, Spain, the United Kingdom, and the United States. Immunosuppressive strategies varied according to therapeutic area and site protocol, and a minority of patients received induction therapy with anti-thymocyte globulin or anti-CD25 monoclonals, while others received maintenance immune suppression that included the calcineurin inhibitors cyclosporine or tacrolimus, purine synthetase inhibitors azathioprine or mycophenolate mofetil, and prednisone. Graft biopsy was performed per protocol or for cause in two and 19 studies, respectively, and histology was interpreted according to the Banff 1997 criteria or subsequent revisions. Two studies examined the prognosis of graft biopsy performed per protocol or for cause within the first year post-transplant (Cosio 2005 [26], Naesens 2013 [46]). Fifteen studies evaluated factors predicting late graft injury, including the anti-HLA antibody (Eng 2011 [35], Fichtner 2016 [36], Gloor 2007 [37], Gosset 2017 [38], Halloran 2016 [39], Courant 2018 [51], Parajuli 2019 [52]), C4d deposition (Kieran 2009 [40], Kikić 2015 [41], Lesage 2015 [42], Moktefi 2017 [44], Sijpkens 2004 [47], Vongwiwatana 2004 [50]), molecular changes in the graft (Loupy 2014 [43]), and treatment (Mulley 2017 [53]). Four studies described the primary importance of TG, the association with DSA and/or C4d deposition, or the potentially confounding influence of hepatitis C virus on the phenotype of graft failure (Cruzado 2011 [34], Moscoso-Solorzano 2010 [45], Sun 2012 [48], Suri 2000 [49]).

**Table 1:** Studies included in the meta-analysis.

Study	Outcome Type	Investigated Contrast	Patients With/Without TG, n	HR (95% CI)	Time Between Treatment and Biopsy, months	Certainty That T(0) Was at Biopsy	Biopsy Type
Cosio 2005 [26]	DCGF	cg > 0 vs. cg = 0	15/87	10 (3.1–34)	12.0	Certain	Protocol
Cruzado 2001 [34]	DCGL	TG vs. recurrence of renal disease, *de novo* glomerulonephritis, chronic allograft nephropathy	11/85	2.98 (1.18–7.56)	66.4	Probable	For cause
Eng 2011 [35]	GL	TG vs. non-TG	61/87	2.85 (1.95–4.16)^a^	50.1	Certain	For cause
Fichtner 2016 [36]	DCGL	TG vs. non-TG	19/43	7.23 (2.46–21.3)	53.5	Certain	For cause
Gloor 2007 [37]	DCGL	TG vs. other types of histological changes	55/527	6.05 (3.15–11.6)	21.0	Probably	For cause^c^
Gosset 2017 [38]	DCGL	TG vs. non-TG	94/1436	4.68 (3.07–7.12)	12	Certain	Protocol
Halloran 2016 [39]	DCGL	cg > 0 vs. cg = 0	94/423	2.4 (1.65–3.48)^a^	No data	Certain	For cause
Kieran 2009 [40]	DCGL	TG vs. non-TG with any other histological changes	19/59	7.7 (3.07–19.30)	161.3	Certain	For cause
Kikić 2015 [41]	DCGL	cg > 0 vs. cg = 0	105/769	1.98 (1.43–2.76)^b^	0.8	Probable	For cause
Lesage 2015 [42]	DCGF	cg > 0 vs. cg = 0	61/61	5.72 (2.73–11.97)	79.0	Certain	For cause
Loupy 2014 [43]	DCGL	cg > 0 vs. cg = 0	Total: 74	1.85 (1.18–2.9)	No data	Probable	For cause
Moktefi 2017 [44]	GL	TG vs. non-TG	16/32	1.04 (0.36–3.01)	22	Certain	For cause
Moscoso-Solorzano 2010 [45]	DCGF	TG vs. interstitial fibrosis/tubular atrophy	37/65	2.9 (1.43–5.88)^a^	31.44	Certain	For cause
Naesens 2013 [46]	DCGL	cg > 0 vs. cg = 0	11/479	8.86 (4.0–19.6)	No data	Certain	For cause
Sijpkens 2004 [47]	DCGF	TG vs. chronic allograft nephropathy without TG	18/108	0.76 (0.36–1.63)^a^	34.8	Certain	For cause
Sun 2012 [48]	DCGL	cg > 0 vs. cg = 0	43/43	2.44 (1.06–5.58)^a^	56.8	Certain	For cause
Suri 2000 [49]	DCGL	TG vs. chronic rejection without TG	25/25	1.89 (1.04–3.44)^a^	65.4	Certain	For cause
Vongwiwatana 2004 [50]	GL	TG vs. recurrent immunoglobulin A nephropathy	31/27	3.2 (1.5–6.84)	78.7	Certain	For cause
Courant 2018 [51]	DCGL	cg > 0 vs. cg = 0	Total: 74	2.71 (1.48–5.00)	25	Certain	For cause
Mulley 2017 [53]	GL	cg > 0 vs. cg = 0	9/15	2.44 (0.73–8.07)	34.2	Certain	For cause^c^
Parajuli 2019 [52]	DCGF	cg > 0 vs. cg = 0	45/542	4.02 (2.28–7.07)	12.3	Certain	For cause^c^

CI, confidence interval; cg, Banff chronic glomerulopathy score; DCGF, death-censored graft failure; DCGL, death-censored graft loss; GL, graft loss; HR, hazard ratio; TG, transplant glomerulopathy; T(0), follow-up initiation.

^a^The data were calculated from Kaplan-Meier curves.

^b^The data were calculated from probability of being event-free at a fixed time point.

^c^Not all biopsy was for cause.

The numbers of patients reported in each study ranged from 24 to 1,530, with a maximum patient follow-up of 35 years at the time of reporting. All but five of the 21 studies provided graft loss–related data as the only end point, either censored for death or not. In the remaining five studies, the combined end points labeled as “death-censored graft failure” included graft loss and some laboratory measure of graft dysfunction—namely, the doubling of serum creatinine levels (Lesage 2015 [42]), >50% reduction in GFR beyond 1 year (Cosio 2005 [26]), GFR <15 mL/min/1.73 m^2^ (Moscoso-Solorzano 2010 [45]), or serum creatinine level ≥150% of the baseline value (Sijpkens 2004 [47]); Parajuli *et al*. [52] did not specify the criteria for failure. Most studies applied explicit censoring for death or death censoring that was deduced from the study design. In four studies (Eng 2011 [35], Moktefi 2017 [44], Vongwiwatana 2004 [50], Mulley 2017 [53]), there was no death censoring, or no clear conclusion on censoring could be made. Quality assessment using the Newcastle-Ottawa Scale concluded that all included studies were of overall “good quality” (S4 Table). In the Selection domain, 19 studies scored the highest mark of 4, and two studies (Courant 2018 [51] and Parajuli 2019 [52]) scored 3. In the Outcome domain, two studies (Eng 2011 [35], Vongwiwatana 2004 [50]) scored 2, and the remainder scored 3.

### Meta-analysis

**Fig 2:**
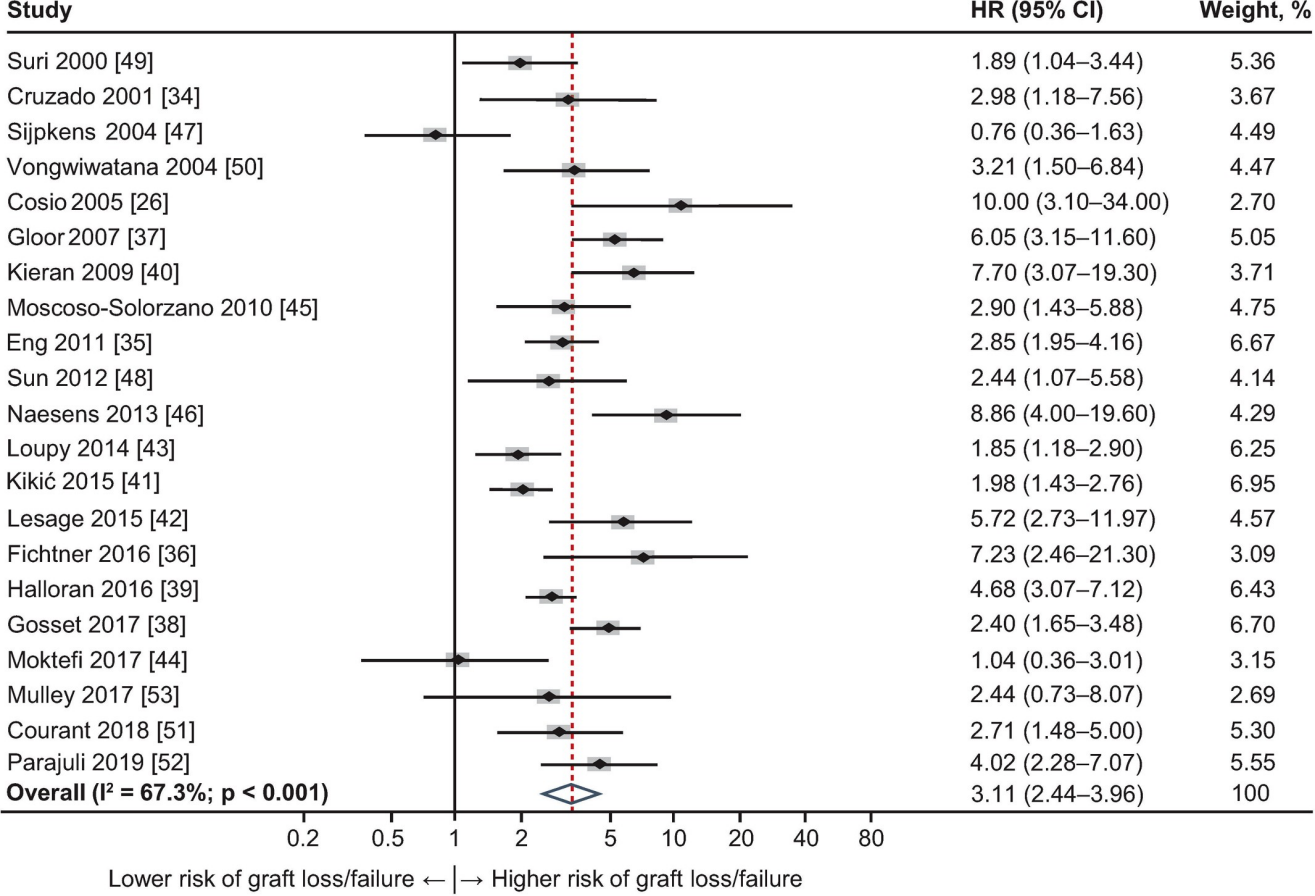
Forest plot of hazard ratios (HRs) for graft loss or failure by presence of transplant glomerulopathy (TG) for studies included in the meta-analysis (n = 21). Weights are from random-effects analysis. CI, confidence interval; HR, hazard ratio.

Random-effects meta-analysis was performed, owing to the heterogeneity of the 21 studies (I^2^ = 67.3%). The combined HR and the individual HRs and weights applied to each study are presented in Fig 2. The combined HR from the random-effects meta-analysis was 3.11 (95% CI 2.44–3.96), indicating that the risk of graft loss or failure was more than threefold higher in patients with TG than in those without TG. In 18 of the 21 studies, the individual HRs indicated a significantly higher risk for graft loss or failure in the group of patients with TG, ranging from 1.85 (95% CI 1.18–2.90) to 10.00 (95% CI 3.10–34.00), whereas in three studies HRs did not (Moktefi 2017 [44], HR = 1.04, 95% CI 0.36–3.01; Sijpkens 2004 [47], HR = 0.76, 95% CI 0.36–1.63; Mulley 2017 [53] HR = 2.44, 95% CI 0.73–8.07). There were no common explanatory features between these three studies with respect to study location, period of observation, or relevant data extracted. There was no significant evidence of publication bias (Egger’s p = 0.18; S1 Fig); that is, there was no evidence that the probability of the findings reported depended on the actual results or size of the study.

To further explore the relationship between TG and graft outcomes, sensitivity analyses were performed within defined subgroups of the studies. Eight such subgroups were constructed as shown in Table 2, according to reason for biopsy, the explicit definition of start of observation period, and patient and graft survival measures reported. Subgroup analyses all demonstrated the association between TG and graft failure/loss to be consistent with the primary analysis, with HRs ranging from 2.58 (95% CI 1.80–3.71) to 4.53 (95% CI 3.19–6.43; Table 2). Within the subgroup where biopsy was reported “for cause” the HR was 2.89 (95% CI 2.20–3.80; I^2^ = 61.6%); where follow-up commenced at the time of biopsy, the HR was 3.24 (95% CI 2.45–4.28; I^2^ = 65.5%); and among studies that censored for death, the HR was 3.30 (95% CI 2.43–4.50; I^2^ = 70.0%).

**Table 2:** Subgroups examined in the sensitivity analyses.

Group	Rationale for Evaluation	Studies, n	I^2^, %	Overall HR (95% CI)
DCGL reported as an outcome	To investigate the effect of TG on loss of graft, where patients who died were excluded from the graft survival analysis	12 [34, 36–41, 43, 46, 48, 49, 51]	70.0	3.30 (2.43–4.50)
DCGF reported (graft loss and other laboratory indicators of graft failure)	To investigate the effect of TG on graft loss/functional deterioration	5 [26, 42, 45, 47, 52]	80.5	3.31 (1.55–7.11)
Graft loss and patient death as outcomes	To investigate the effect of TG in studies that did not censor death	4 [35, 44, 50, 53]	11.4	2.58 (1.80–3.71)
Studies that included published HRs and CIs	To investigate the effect of TG in studies that presented HR and CIs, avoiding uncertainties around the estimation of the HR	13 [26, 34, 36–38, 40, 42–44, 46, 51–53]	62.7	4.11 (2.94–5.73)
Follow-up reported as commencing at time of biopsy	To investigate the effect of TG in studies that defined follow-up initiation at the time of biopsy	17 [26, 35, 36, 38–40, 42, 44–53]	65.5	3.24 (2.45–4.28)
Follow-up reported as commencing at time of biopsy and published HR	To investigate the effect of TG in studies that met the strict criteria where follow-up was reported as commencing at the time of biopsy and HR was published	10 [26, 36, 38, 40, 42, 44, 46, 51–53]	50.3	4.53 (3.19–6.43)
For-cause biopsy and time to biopsy data reported	To investigate the effect of TG in studies that were used in the meta-regression analysis	16 [34–37, 40–42, 44, 45, 47–53]	61.6	2.89 (2.20–3.80)
For-cause biopsy reported, DC graft outcome and time to biopsy data	To investigate the effect of TG in a subgroup of studies used in the meta-regression analysis that censored death	12 [34, 36, 37, 40–42, 45, 47–49, 51, 52]	69.1	3.09 (2.18–4.37)

CI, confidence interval; DC, death-censored; DCGF, death-censored graft failure; DCGL, death-censored graft loss; HR, hazard ratio; TG, transplant glomerulopathy.

### Meta-regression

**Fig 3:**
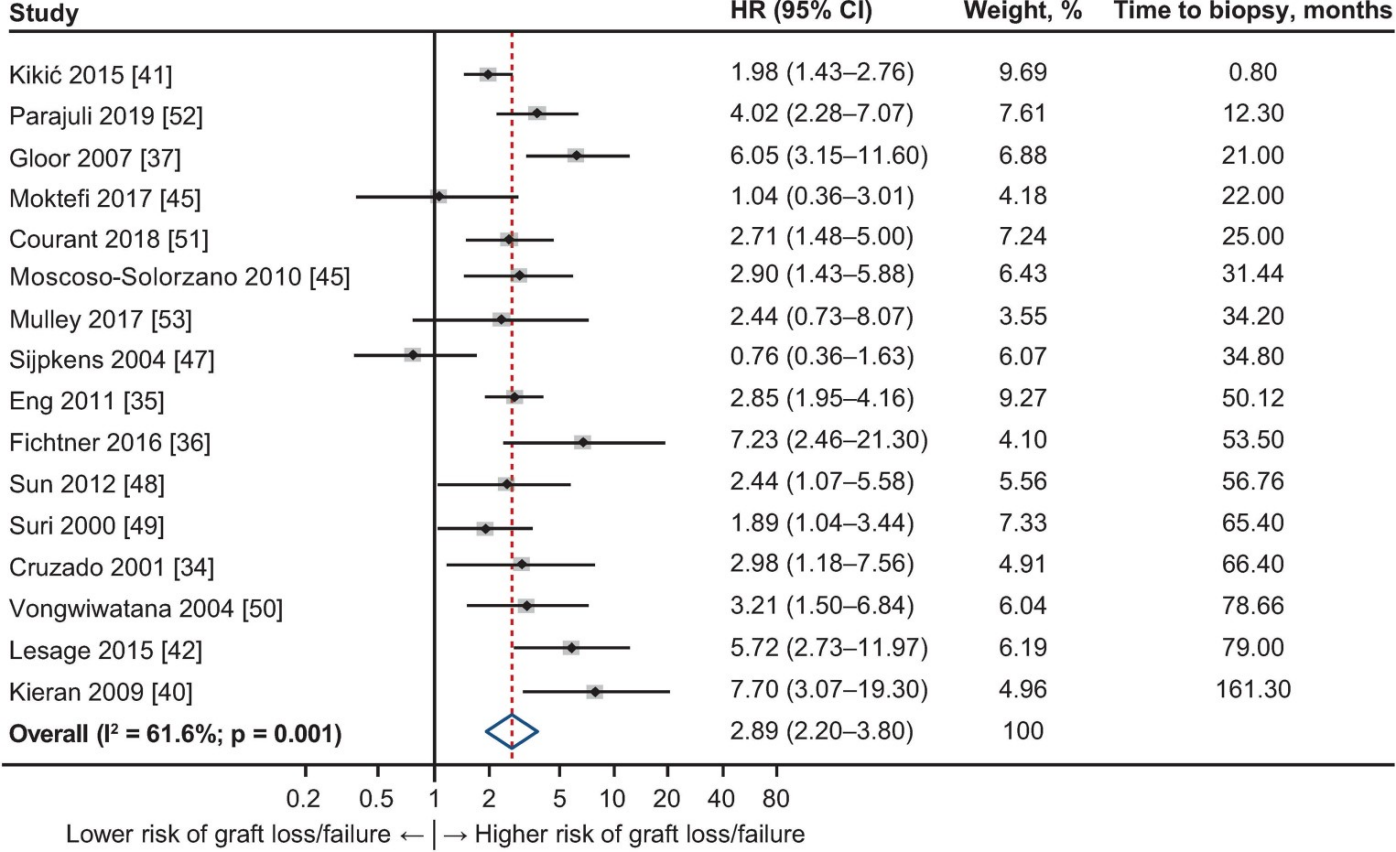
Forest plot of hazard ratios (HRs) for graft loss or failure for studies with for-cause biopsy and data with time to biopsy from transplantation, ordered by time to biopsy (n = 16). Weights are from random-effects analysis. CI, confidence interval.

Meta-regression analysis was conducted to examine other factors that might influence the relationship between TG and graft outcome. Neither age at diagnosis or at transplantation, reported in 19 articles (HR = 0.994, 95% CI 0.955–1.035; p = 0.77), nor recipient sex, reported in 15 articles (HR = 1.024, 95% CI 0.967–1.084; p = 0.39), had a significant effect on the relationship observed. Analysis using the study-specific mean time from transplantation to biopsy as the independent variable and the logarithm of HR as the dependent variable, using the 16 studies in which the time to for-cause biopsy was specified (range, 0.8–161.3 months), demonstrated a trend toward an increase in risk of graft failure as this time increased (HR = 1.007, 95% CI 0.998–1.015), although this effect did not reach statistical significance (p = 0.12; Fig 3). Other covariates such as donor source or physiological status, or recipient factors of diabetes or hypertension, were not available within the data set.

### Median overall graft survival time in patients with or without TG

**Fig 4:**
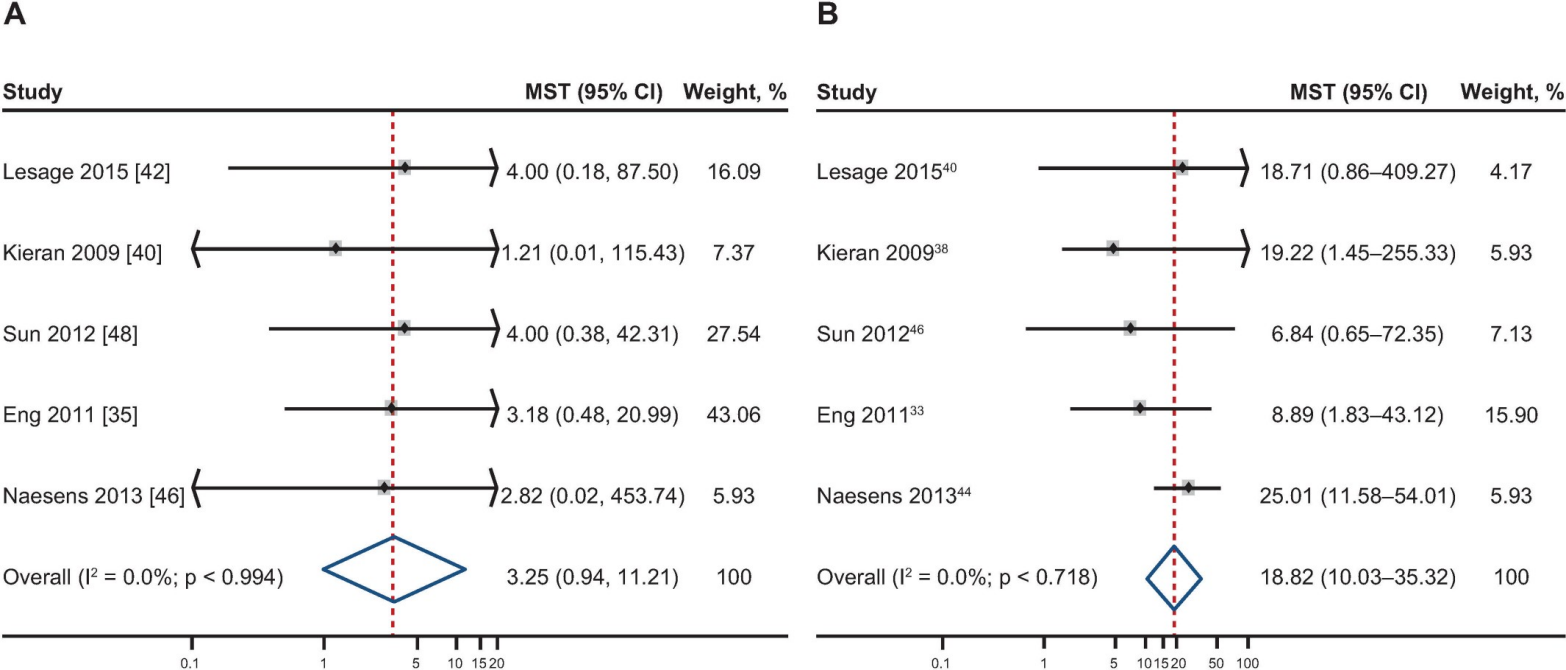
Individual and pooled median survival times (MSTs; year) with 95% confidence intervals (CIs). (A) Including the relative weight of each transplant glomerulopathy (TG) study group. (B) Including the relative weight of each non-TG study group. Graft follow-up data started at the time of diagnostic biopsy. Weights are from random-effects analysis.

Median overall graft survival time was estimable for the TG and non-TG groups from five studies (Eng 2011 [35], Kieran 2009 [40], Lesage 2015 [42], Naesens 2013 [46], Sun 2012 [48]; Table 3). Individual and pooled median survival times are presented in Fig 4A for patient groups with TG and Fig 4B for patient groups without TG, with graft follow-up data beginning at the time of diagnostic biopsy. Individual median overall survival times ranged between 1.21 and 4.00 years in the TG groups, and between 6.84 and 25.01 years in the non-TG groups. Pooled analysis showed that the median overall survival time among patients with TG was 3.25 (95% CI 0.94–11.21) years, approximately 15 years shorter than in patients without TG (18.82 [95% CI 10.03–35.32] years).

**Table 3:** Studies with their meta-analysis input data (number of patients and MST) and source of MST.

Study	Input data^a^			
	TG		Non-TG	
	Patients, n	MST, years (source)	Patients, n	MST, years (source)
Lesage 2015 [42]	61	4.00 (reported)	61	18.71 (estimated with Weibull model)
Kieran 2009 [40]	19	1.21 (read off from KM curve)	59	19.22 (estimated with Weibull model)
Sun 2012 [48]	43	4.00 (read off from KM curve)	43	6.84 (estimated with Weibull model)
Eng 2011 [35]	61	3.18 (read off from KM curve)	87	8.89 (read off from KM curve)
Naesens 2013 [46]	11	2.82 (read off from KM curve)	479	25.01 (estimated with Weibull model)

KM, Kaplan-Meier; MST, median survival time; TG, transplant glomerulopathy.

^a^Graft follow-up data started at the time of diagnostic biopsy.

## Discussion

TG is a morphological lesion resulting from continuing or repetitive endothelial cell injury in the renal graft, which is a harbinger of graft failure [13, 54]. Initiated most frequently by binding of recipient antibodies to donor HLA or other molecular targets, downstream inflammation is amplified by complement activation, the release of soluble mediators, and the involvement of numerous inflammatory cell types, including monocytes/macrophages, lymphocytes, natural killer cells, and neutrophils [13, 20]. Ultrastructural changes include endothelial cell swelling or vacuolization, loss of endothelial fenestrations, subendothelial widening of the lamina rara interna with electron-lucent or flocculent material, and reduplication or multilamination of the lamina densa [13]. TG may also occur less frequently with cell-mediated rejection, thrombotic microangiopathy, or hepatitis C virus infection, reflecting a common pathway of EI and vascular remodeling [22]. TG is associated with reduced podocyte density and proteinuria, and typically progresses to irreversible reduction in glomerular filtration and graft loss [55].

This first comprehensive systematic review and meta-analysis confirms that TG is an important histological marker of impending graft failure. Quantitative analysis shows that the probability of graft failure is increased more than threefold when TG is documented on routine or for-cause graft biopsy, and that failure occurs within a mean duration of 3 years compared with 18 years in patients without this histological finding. While the historical data available to this point do not permit more precise stratification of risk, the recent analysis of more site-restricted data by these results provide important support for TG as a robust surrogate marker for trials exploring innovative therapeutic strategies to prevent or treat AMR, as an index of caution to avoid over-treatment when no proven therapy is available to reverse this lesion, and as a clinical indicator for the implementation of structured management strategies to prepare for safe transition to dialysis.

Despite the recognition of TG as a serious prognostic indicator [13], reports enabling objective evaluation of its quantitative influence remain relatively scarce. Of more than 5,000 publications reviewed, only 21, comprising just over 5,800 evaluable patients, provided comparative data suitable for analysis. These reports were heterogeneous in geographic location, timing, period of observation, study population, sample size, study purpose, treatment, reason for biopsy, precise outcome measures, and other factors. Seven studies reported data from North America, nine reported data from Europe, and one study each reported data from Asia, Australia, and Latin America; biopsy was performed for cause in almost 90% of studies, and two-thirds of studies examined causative or diagnostic parameters associated with AMR or TG (e.g. circulating DSA, C4d deposition, or transcriptomic evidence of molecular injury). Almost 90% of these studies showed a higher risk of graft loss or failure among patients with TG. Sensitivity analyses, conducted to explore the direction and magnitude of the effect of TG within report clusters defined according to biopsy rationale, overall or death-censored graft loss, or other factors, showed HRs ranging from 2.58 to 4.53, supporting the overall HR of 3.11 observed in the full analysis.

Only five of the reports provided graft survival data enabling the estimation of survival time in patients with or without TG as an indicator of absolute time to graft loss/failure. All reports were consistent, however, and median survival time values were 1.21–4.00 years for patients with TG compared with 6.84–25.01 years for those without this lesion. This close association between TG and both quantitative risk of graft loss and time to event is consistent with other reports that did not provide robust data, enabling direct comparison between histological groups required for inclusion in this meta-analysis [55].

The heterogeneity of the data reported limited the ability to explore additional risk factors, which may influence the relationship between the presence of TG and graft outcome. The observed effects were not explained by patient age or sex, both factors reported as being associated with increased graft failure risk [56–59], although the inclusion of studies comprising mostly adult patients may have skewed the findings with respect to age in this meta-analysis. TG may be detected early or late post-transplant, and mechanisms of vascular injury and remodeling may evolve over time, with acute endothelial inflammation leading to progressive podocyte depletion and reduced allograft function [55]. Although insufficient information was contained in the reports to explore this in detail, meta-regression analyses evaluating time to biopsy from transplant showed that the effect of TG on the risk of graft loss or failure increased as time elapsed, although this trend did not reach statistical significance.

Due to the paucity of data, we were unable to quantitate the influence of immune measures on graft outcomes. The presence of DSAs has been reported as an important factor in the development and progression of TG, and several of the studies analyzed supported this association [35, 37–39]. Activation of the complement cascade may contribute to the inflammatory EI, and certain reports indicated accelerated graft loss in patients with C1q-fixing DSAs [36] or peritubular deposition of C4d [40–42, 44, 47, 48, 50]. These observations are consistent with the systematic review and meta-analysis reported by Bouquegneau *et al*. [23], confirming the increased risk of rejection and graft loss conferred by the presence of complement-binding DSAs, although other studies reported contrasting findings [44]. Sub-phenotyping of inflammation according to the combination of microcirculatory injury and glomerulopathy and the identification of endothelial transcripts on gene expression analysis may further improve risk prediction [39]. Together, these observations suggest that continuing active humoral injury potentiates vascular remodeling and accelerates graft failure. However, it is evident that TG may be detected in the absence of DSAs, perhaps reflecting the relapsing nature of the immune injury [60]. Hepatitis C virus may be associated with TG, and appeared as a risk factor for graft loss in two of the studies analyzed here [34, 45], although whether this was due to progression of the specific lesion was uncertain.

Many of these anticipated risks have now been confirmed by the superb analysis recently published by Aubert *et al*. examining data compiled by four sites in Paris and Canada [61]. This study used a probabilistic archetype analysis of 385 patients with biopsy-proven TG from 2004 to 2014, combining comprehensive pathology findings with clinical, immunological, and outcome data to identify distinct patient groups. Median time from transplant to biopsy diagnosis of TG was 33 months, and graft survival was 57% and 25%, respectively, at 5 and 10 years post-diagnosis. Within this framework Aubert *et al*. distinguished five recipient groups ranging from Archetype 1, with the best-preserved GFR (53 ± 25 mL/min/1.73 m^2^), the lowest histological change as measured by the Banff chronic glomerulopathy score, and only low-grade proteinuria, to Archetype 5, with the highest proportion of prior transplants and patients with circulating DSA; diffuse and severe histological change with a high microvascular inflammatory burden and C4d at the time of TG diagnosis; and more frequent use of plasma exchange, intravenous immunoglobulin, eculizumab, and bortezomib for AMR. Graft survival declined from 88% to 22% across the 5 archetypes at 5 years of follow-up.

### Limitations

This meta-analysis is subject to certain limitations, and to minimize these, we conducted a risk of bias assessment to evaluate the quality of the included studies. Because only cohort studies were included, quality assessment required an instrument suitable for use with nonrandomized studies. We selected the widely-used Newcastle-Ottawa Scale for this rather than the more recent GradePro, and assessed the quality of studies using two of the three domains, Selection and Outcome. Small anecdotal reports with extremely limited numbers of cases, from which valid comparative information could not be derived, were considered to be non-contributory and were excluded. While this may potentially influence selection, our detailed review showed that the threshold of a minimum of 10 cases provided a simple and clear minimum for such articles. Certain studies did not provide granular data, including HRs or number of events, which instead were estimated using published Kaplan-Meier curves, and censoring may have influenced the number of events and standard errors estimated from the study reports. In the absence of the original data sets, appropriate methodology was used to allow these studies to be included in the analysis. However, the sensitivity analysis showed that if only studies with published HRs and CIs were included, the results were consistent with the primary analysis.

## Conclusions

Despite the limitations, the study provides robust evidence that the histological diagnosis of TG is associated with a more than threefold risk of graft failure or loss, which occurs within a short time following diagnosis, resulting in a more than fivefold reduction in expectation of graft survival. These data underscore the importance of preventing the onset of TG following kidney transplant and provide a foundation for considering the use of TG as a viable marker in studies designed to prevent or reverse chronic AMR. Current studies conducted by the Genome Canada Transplant Consortium will enable more precise definition of the probabilities and timelines of graft failure in patients with TG within the context of current immunosuppression and medical care. These data will then be incorporated as a robust outcome measure for use in prospective therapeutic trials, and as a guide to the management of patients with graft failure following transplantation.

## Supporting information

S1 Table Search strategy terms used in the systematic literature review. (DOCX)

S2 Table Search engine approach used in the systematic literature review. (DOCX)

S3 Table Reasons for exclusion of articles from the median survival time meta-analysis. (DOCX)

S4 Table Risk of bias assessment with the Newcastle-Ottawa Scale. (DOCX)

S1 Fig Funnel plot of study publication bias. (DOCX)

S1 File PRISMA 2009 checklist. (DOC)
