# Supplementary material for: Association between transplant glomerulopathy and graft outcomes following kidney transplantation: A meta-analysis
Source: PLoS One. 2020 Apr 28;15(4):e0231646. doi: 10.1371/journal.pone.0231646 (PMC7188300; doi:10.1371/journal.pone.0231646)
Supplement: S1 Table — (DOCX) [file pone.0231646.s001.docx]

**S1 Table. Search strategy terms used in the systematic literature review.**

| **No.** | **Terms** |
| --- | --- |
| #1 | “Transplant” and “glomerulopathy” |
| #2 | “Chronic” and “glomerulopathy” |
| #3 | “Allograft” and “glomerulopathy” |
| #4 | “cg” and “score” |
| #5 | “Banff” and “score” |
| #6 | “cg0” |
| #7 | “cg1” |
| #8 | “cg2” |
| #9 | “cg3” |
| #10 | “cABMR” |
| #11 | “cAMR” |
| #12 | “Chronic” and “antibody” and “mediated” and “rejection” |
| #13 | **#1 OR #2 OR #3 OR #4 OR #5 OR #6 OR #7 OR #8 OR #9 OR #10 OR #11 OR #12** |
| #14 | “Survival” |
| #15 | “Failure” |
| #16 | “Loss” |
| #17 | “Lost” |
| #18 | “Rejection” |
| #19 | **#14 OR #15 OR #16 OR #17 OR #18** |
| #20 | **#13 AND #19** |

cg, Banff chronic glomerulopathy score; cABMR, chronic antibody-mediated rejection; cAMR, chronic antibody-mediated rejection.
