# Supplementary material for: Association between transplant glomerulopathy and graft outcomes following kidney transplantation: A meta-analysis
Source: PLoS One. 2020 Apr 28;15(4):e0231646. doi: 10.1371/journal.pone.0231646 (PMC7188300; doi:10.1371/journal.pone.0231646)
Supplement: S2 Table — (DOCX) [file pone.0231646.s002.docx]

**S2 Table. Search engine approach used in the systematic literature review.**

| **Search Engine** | **Databases** | **Limitations** | **Comment** |
| --- | --- | --- | --- |
| Scopus | No limitations | To title, abstract, keyword  To English language publications |  |
| EBSCO | Academic Search Complete, CINAHL | To title, abstract, subject term  To English language publications | Automatic removal of exact duplicates |
| Cochrane Library | Cochrane Database of  Systematic Reviews 2005 to 3 July, 2019,  ACP Journal Club 1991 to June 2019,  Database of Abstracts of  Reviews of Effects First  Quarter 2016,  Cochrane Central Register of Controlled Trials June, 2019,  Cochrane Methodology  Register Third Quarter 2012,  Health Technology  Assessment Fourth Quarter 2016,  NHS Economic Evaluation Database First Quarter 2016 | No limitations |  |

ACP, American College of Physicians; NHS, National Health Service (UK).
