# Supplementary material for: Association between transplant glomerulopathy and graft outcomes following kidney transplantation: A meta-analysis
Source: PLoS One. 2020 Apr 28;15(4):e0231646. doi: 10.1371/journal.pone.0231646 (PMC7188300; doi:10.1371/journal.pone.0231646)
Supplement: S3 Table — (DOCX) [file pone.0231646.s003.docx]

**S3 Table. Reasons for exclusion of articles from the median survival time meta-analysis.**

| Study | Reasons |
| --- | --- |
| Cosio 2005 [1] | No observed event in the non-TG group |
| Cruzado 2001 [2] | No single comparative group |
| Fichtner 2016 [3] | No relevant KM curve that can be digitized, no reported MST |
| Gloor 2007 [4] | No relevant KM curve that can be digitized, no reported MST |
| Gosset 2017 [5] | No relevant KM curve that can be digitized, no reported MST |
| Halloran 2016 [6] | No single comparative group |
| Kikić 2015 [7] | No relevant KM curve that can be digitized, no reported MST |
| Loupy 2014 [8] | No relevant KM curve that can be digitized, no reported MST |
| Moktefi 2017 [9] | No relevant KM curve that can be digitized, no reported MST in the TG vs. non-TG contrast |
| Moscoso-Solorzano 2010 [10] | TG patients were compared with a group with other pathology than TG |
| Sijpkens 2004 [11] | TG patients were compared with a group with other pathology than TG |
| Suri 2000 [12] | TG patients were compared with a group with other pathology than TG |
| Vongwiwatana 2004 [13] | TG patients were compared with a group with other pathology than TG |
| Courant 2018 [14] | No relevant KM curve that can be digitized, no reported MST |
| Mulley 2017 [15] | No relevant KM curve that can be digitized, no reported MST |
| Parajuli 2018 [16] | No relevant KM curve that can be digitized, no reported MST |

KM, Kaplan-Meier; MST, median survival time; TG, transplant glomerulopathy.

**References**

1. Cosio FG, Grande JP, Wadei H, Larson TS, Griffin MD, Stegall MD. Predicting subsequent decline in kidney allograft function from early surveillance biopsies. Am J Transplant. 2005;5(10): 2464–2472. doi: 10.1111/j.1600-6143.2005.01050.x.

2. Cruzado JM, Carrera M, Torras J, Grinyo JM. Hepatitis C virus infection and de novo glomerular lesions in renal allografts. Am J Transplant. 2001;1(2): 171–178.

3. Fichtner A, Süsal C, Höcker B, Rieger S, Waldherr R, Westhoff JH, et al. Association of C1q-fixing DSA with late graft failure in pediatric renal transplant recipients. Pediatr Nephrol. 2016;31(7): 1157–1166. doi: 10.1007/s00467-016-3322-8.

4. Gloor JM, Sethi S, Stegall MD, Park WD, Moore SB, DeGoey S, et al. Transplant glomerulopathy: Subclinical incidence and association with alloantibody. Am J Transplant. 2007;7(9): 2124–2132. doi: 10.1111/j.1600-6143.2007.01895.x.

5. Gosset C, Viglietti D, Rabant M, Verine J, Aubert O, Glotz D, et al. Circulating donor-specific anti-HLA antibodies are a major factor in premature and accelerated allograft fibrosis. Kidney Int. 2017;92(3): 729–742. doi: 10.1016/j.kint.2017.03.033.

6. Halloran PF, Merino Lopez M, Barreto Pereira A. Identifying subphenotypes of antibody-mediated rejection in kidney transplants. Am J Transplant. 2016;16(3): 908–920. doi: 10.1111/ajt.13551.

7. Kikić Ž, Kainz A, Kozakowski N, Oberbauer R, Regele H, Bond G, et al. Capillary C4d and kidney allograft outcome in relation to morphologic lesions suggestive of antibody-mediated rejection. Clin J Am Soc Nephrol. 2015;10(8): 1435–1443. doi: 10.2215/cjn.09901014.

8. Loupy A, Lefaucheur C, Vernerey D, Chang J, Hidalgo LG, Beuscart T, et al. Molecular microscope strategy to improve risk stratification in early antibody-mediated kidney allograft rejection. J Am Soc Nephrol. 2014;25(10): 2267–2277. doi: 10.1681/asn.2013111149.

9. Moktefi A, Parisot J, Desvaux D, Canoui-Poitrine F, Brocheriou I, Peltier J, et al. C1q binding is not an independent risk factor for kidney allograft loss after an acute antibody-mediated rejection episode: A retrospective cohort study. Transpl Int. 2017;30(3): 277–287. doi: 10.1111/tri.12905.

10. Moscoso-Solorzano G, Camara NO, Franco MF, Araujo S, Ortega F, Pacheco-Silva A, et al. Glomerular damage as a predictor of renal allograft loss. Braz J Med Biol Res. 2010;43(6): 557–564.

11. Sijpkens YW, Joosten SA, Wong MC, Dekker FW, Benediktsson H, Bajema IM, et al. Immunologic risk factors and glomerular C4d deposits in chronic transplant glomerulopathy. Kidney Int. 2004;65(6): 2409–2418. doi: 10.1111/j.1523-1755.2004.00662.x.

12. Suri DL, Tomlanovich SJ, Olson JL, Meyer TW. Transplant glomerulopathy as a cause of late graft loss. Am J Kidney Dis. 2000;35(4): 674–680.

13. Vongwiwatana A, Gourishankar S, Campbell PM, Solez K, Halloran PF. Peritubular capillary changes and C4d deposits are associated with transplant glomerulopathy but not IgA nephropathy. Am J Transplant. 2004;4(1): 124–129.

14. Courant M, Visentin J, Linares G, Dubois V, Lepreux S, Guidicelli G, et al. The disappointing contribution of anti-human leukocyte antigen donor-specific antibodies characteristics for predicting allograft loss. Nephrol Dial Transplant. 2018;33(10): 1853–1863. doi: 10.1093/ndt/gfy088.

15. Mulley WR, Huang LL, Ramessur Chandran S, Longano A, Amos LAR, Polkinghorne KR, et al. Long-term graft survival in patients with chronic antibody-mediated rejection with persistent peritubular capillaritis treated with intravenous immunoglobulin and rituximab. Clin Transplant. 2017;31(9). doi: 10.1111/ctr.13037.

16. Parajuli S, Joachim E, Alagusundaramoorthy S, Aziz F, Blazel J, Garg N, et al. Donor-specific antibodies in the absence of rejection are not a risk factor for allograft failure. Kidney Int Rep. 2019;4(8): 1057–1065. doi: 10.1016/j.ekir.2019.04.011.
