# Supplementary material for: Association between transplant glomerulopathy and graft outcomes following kidney transplantation: A meta-analysis
Source: PLoS One. 2020 Apr 28;15(4):e0231646. doi: 10.1371/journal.pone.0231646 (PMC7188300; doi:10.1371/journal.pone.0231646)
Supplement: S4 Table — (DOCX) [file pone.0231646.s004.docx]

**S4 Table. Risk of bias assessment with the Newcastle-Ottawa Scale.**

| **Studies** | **Number of stars in the domains** | | **Overall quality** |
| --- | --- | --- | --- |
|  | **Selection** | **Outcome** |  |
| Cosio 2005 [1] | 4 | 3 | Good quality |
| Cruzado 2001 [2] | 4 | 3 | Good quality |
| Eng 2011 [3] | 4 | 2 | Good quality |
| Gloor 2007 [4] | 4 | 3 | Good quality |
| Kieran 2009 [5] | 4 | 3 | Good quality |
| Kikic 2015 [6] | 4 | 3 | Good quality |
| Lesage 2015 [7] | 4 | 3 | Good quality |
| Loupy 2014 [8] | 4 | 3 | Good quality |
| Moscoso-Solorzano 2010 [9] | 4 | 3 | Good quality |
| Naesens 2013 [10] | 4 | 3 | Good quality |
| Sijpkens 2004 [11] | 4 | 3 | Good quality |
| Sun 2012 [12] | 4 | 3 | Good quality |
| Suri 2000 [13] | 4 | 3 | Good quality |
| Vongwiwatana 2004 [14] | 4 | 2 | Good quality |
| Fichtner 2016 [15] | 4 | 3 | Good quality |
| Gosset 2016 [16] | 4 | 3 | Good quality |
| Halloran 2016 [17] | 4 | 3 | Good quality |
| Moktefi 2017 [18] | 4 | 3 | Good quality |
| Courant 2018 [19] | 3 | 3 | Good quality |
| Mulley 2017 [20] | 4 | 3 | Good quality |
| Parajuli 2018 [21] | 3 | 3 | Good quality |
