# Supplementary material for: Association between transplant glomerulopathy and graft outcomes following kidney transplantation: A meta-analysis
Source: PLoS One. 2020 Apr 28;15(4):e0231646. doi: 10.1371/journal.pone.0231646 (PMC7188300; doi:10.1371/journal.pone.0231646)

**S1 Fig. Funnel plot of study publication bias.** There is no "white area" in the left lower section of the plot where the studies with a low patient number and small effects would have been expected (n = 21). lnHR, logarithm of hazard ratio, SE, standard error.


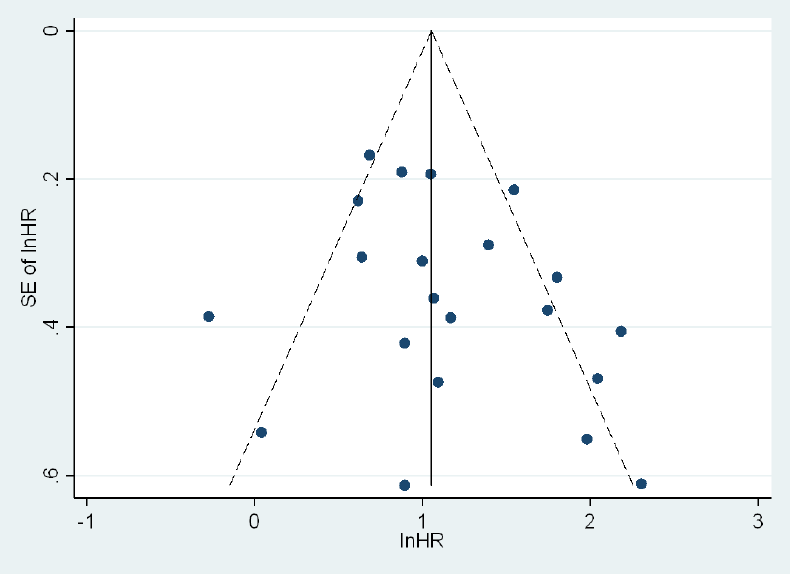

Supplement: S1 Fig — (DOCX) [file pone.0231646.s005.docx]
